# Problems of Social Hygiene

**Published:** 1913-11

**Authors:** Theo. Y. Hull

**Affiliations:** Secretary State Society of Social Hygiene


### Department of Eugenics.

Conducted by Dr. Malone Duggan and Dr. Theo. Y. Hull.

**THE TEXAS STATE SOCIETY OF SOCIAL HYGIENE.**

Headquarters: San Antonio, Texas.

DR. MALONE DUGGAN, President, San Antonio.
DR. F. E. DANIEL, 1st Vice President, Austin.
PROF. A. CASWELL ELLIS, 2d Vice President, Austin.
MRS. ELI HERTZBERG, 3d Vice President, San Antonio.
DR. THEO. Y. HULL, Secretary, San Antonio.
MR. W. L. EVANS, Treasurer, San Antonio.

EXECUTIVE COMMITTEE.

Dr. Malone Duggan, Chm.
Dr. Theo. Y. Hull, Sec'y.
Dr. W. F. McCaleb.
Hon. C. H. Jenkins.
Rev. A. W. S. Garden.
Hon. J. C. Willacy.
Mr. W. L. Evans.
Dr. Frank D. Boyd.
Dr. J. R. Nichols.
Dr. J. M. McCutchan.
Dr. W. A. King.
Dr. J. W. Torbett.
Dr. F. C. Walsh.
Dr. Joe Reuss.
Dr. Frank Paschal.

### Problems of Social Hygiene.

BY THEO. Y. HULL, M. D., SECRETARY STATE SOCIETY OF SOCIAL HYGIENE.

Most of the great world movements for social betterment occur periodically. They are based upon some great public need. A condition arises, continues to grow worse through many generations, until it threatens dire consequences to the race unless its progress can be checked. The continued presence of an evil, if sufficiently grave, stimulates antagonistic forces, which in time overcome the evil. The stimulus withdrawn, the public sinks to rest, while a new enemy is approaching with insidious step.

The present stiuation which has given rise to the social hygiene movement is not of recent origin. It is as old as civilization itself. Indeed it was regarded for centuries as a concomitant of civilization —a necessary evil—and is so regarded today by a pretty large per cent of the human race. Through all these centuries the tortuous trail of the serpent of social disease is visible with ever tightening grip upon the race.

It is only in the last generation that the social body has realized that the continuance of social disorder means social degeneration. It is a still shorter time since the social body realized the possibility of emancipation from the deterrent effects of ages of accumulated vice. It is only now that a determined effort to arouse the national conscience is being carried out. The great movement is still in the hands of the comparatively few but valient workers.

Only a few years ago the great wave, or what some may term clamor, for the conservation of public resources swept over the country. It followed the realization that the country must suffer from the continued waste and monopolization of government forests, mineral deposits and sites for the utilization of the great water power of the country.

In this case the stimulus was applied to the protection of the financial and commercial interest of the people, but the aroused public began to realize that there were other interests of a deeper and more fundamental character at stake, that there was something that made property and commerce of value, and that that something was human life. The movement begun for the conservation of natural resources has supplied the stimulus for the conservation of the greatest of the national assets, the lives and health of the men and women and their children that constitute the nation, the conservation of human power.

The attempt to arouse the social body to this phase of the question of conservation has called for the solution of a new set of social problems classed under the general phrase, social hygiene. The basic problems of this movement are those of *information, education* and *legislation.*

Before the movement could make headway, definite information must be obtained. There is always a great mass of hearsay evidence floating around in the world, but this will not often bear scientific scrutiny. So the first work was to collect and classify the material that must be used in the active crusade. A great deal has been written on this and that subject, some of which was true and some merely the expressed belief. Some of the writers, like Fournier and Morrow, had large experience on which to base their conclusion. Other writers of less extensive experience had expressed their opinions drawn from their own observation. The first problem, therefore, was to collect and sift out the chaff and when this was done, to classify what remained, for some of it related to the prevalence of prostitution, some to the occurrence of the social diseases and their effect upon the public health, and still some others related to the laws of inheritance in man. Scratch beneath the surface of the social body wherever they would, the evidence of social crimes and concomitant evils was manifest.

The very first thing that came to light was the enormity of the crime of prostitution. Every one at all conversant with the subject knew that this great plague was sapping the strength of the people, but a few were prepared for the revelations brought out by systematic inquiry and investigation. The reports of the actual condition in cities like New York, Chicago and Baltimore are hard for the uninitiated in such matters to believe. Investigation in other countries revealed the same startling condition, varying only with local conditions. Prostitution, therefore, is the burning question of this day, as it has been of former days; for the evidence is positive that wherever it exists it saps the vitality of the people, and decadence, individual and national, is inevitable. If we were to estimate the cost of immorality to the public, we would find an enormous waste running up into the hundreds of millions. The money waste caused by the prevalence of prostitution is the one item in the cost of crime that, owing to our highly developed commercial instincts, we are able to estimate quite accurately. But it is only one of the wastes. Dr. Howard A. Kelly, in speaking of the enormous waste revealed by these investigations, said, "But investigations can not reveal or tabulate, or even faintly suggest, the infinite loss in the incalculable sum of human suffering, sorrow, misery, degradation, disappointed hopes, family tragedies; it can not sum up the wrecked lives of several millions of men and women and children, nor the enormous and increasing sterility which alone is alarming from the standpoint of national conservation."

But the evils of commercialized vice, for prostitution is largely a matter of dollars and cents, in its last analysis, does not stop with degradation, disappointed hopes, family tragedies and wrecked lives. It debauches everything it touches and reaches out into the future for generations.

The prevalence of the social diseases was known to be great, but few were prepared for the enormous number of human beings in this "land of the free and home of the brave" known to be infected. The investigation shows that incredible numbers of young men become infected every year, only to carry these vile diseases into their married life to reap a most unwonted harvest. It is said that the evil that men do lives after them. In regard to the victims of the social diseases this is a mild way to put it, for this evil not only lives on, but it keeps on destroying lives and morals. We see one of them putting out the eyes of the young and creating a world of darkness and horror for them. We see it again destroying the highest functions in the body in thousands upon thousands of those who should be the country's fathers and mothers. We see another disease destroying the young by thousands and tens of thousands. We see the same disease striking down, often in the very prime of life, its thousands (apoplexy 60,000) and rendering halt still other thousands (locomotor ataxia). We see it again filling our almshouses, our prisons, and our insane asylums with an ever increasing multitude of those that fall by the wayside. The investigation gives figures, but the figures are immaterial—we can not appreciate them.

But prostitution and the social diseases are not all the investition has studied. It has looked into the future. For commercial purposes, man has long studied the sequence of inheritance, but when applied to himself he had no time to consider it. It was all right to talk of pedigrees in plants and animals that may be turned to financial account, but the highest of all animals, man, was a separate being, distinct and apart. But science has made known that inheritance in man obeys the same laws as other living beings. The main great facts in inheritance stand out so that even those that run may read, and even the blind may profit by it.

This much the study of the first problem of the social hygiene movement has revealed. Most rational beings will agree that the information is sufficient. The charges are more than proven. What was belief is now certainty. The piling up of evidence can only increase the quantity, but not alter the kind. Thus the desired information is at hand. The other problems relate to its application.

The second problem of the social hygiene movement is that of *education*. A movement of this kind must appeal to the intelligence of the people. It must awaken the public conscience, as Sir Francis Galton said, like a new religion. It must allay prejudice and overcome long established customs. This can not be accomplished in a day. It will take years to educate the public in the higher ideals.

The second problem, therefore, is one of public enlightenment. It is how to use the material at our command in such a way as not to arouse prejudice and at the same time appeal to the common sense of the people. For, after all, it is the common sense application of information that counts most largely in progress.

The problem of education includes many questions. It includes the question of prostitution in all its phases of public, clandestine and commercialized vice. For centuries the public has seemingly refused to believe in this hydra-headed monster of iniquity, and the lower strata of officialdom have joined hands with it in destroying public morals and private health, while the higher officials have considered it out of their domain. In all this the few have waged the battle and the many have looked on but have not seen.

The aroused public interest and the broader intelligence of the present make this an opportune time for the dissemination of the information gathered by hundreds of observers. If once the real public, not the official public, can be reached and interested, the solution of the question will be at hand.

The problem of education also includes the dissemination of the evidences relative to the prevalence of the social diseases, and the influence they bear on public health, on public and individual efficiency, on mental deficiency, on criminality, on poverty, and on all grades and forms of insanity and moral worthlessness. If one of these diseases is present in not less than five per cent of the people, and its existence entails a long series of consequences not only to the infected person but to many others, the public should be made aware of the truth, for the public must bear the consequences ultimately. If locomotor ataxia and apoplexy and general paresis kill more people each year than compose our national army, the people should know the chief factor in this wholesale destruction. If even an appreciable per cent of our unpardonable infant mortality, or infant murder, is due to the same cause, it should be made known. If ten per cent of all sickness, and many say more, is due to the same evil, the public should know it. If insanity, degeneracy and incompetency for the same reasons are increasing at anything like the rate our statistics show, this fact should be made known that the intelligent people may act.

If another one of these diseases which have their source in commercialized vice puts out the eyes of thousands of infants, makes invalids of tens of thousands of women, and causes practically every seventh home to be childless, this should be made known. Lesser evils have involved nations in mad conflicts. It is a matter of grave concern; for nations and States and municipalities are composed of men and women and their offspring, and no State can rise higher than its people.

This information must be forced home in some way, if only by repetition and reiteration, until national and State and municipal bodies take cognizance of it and act for the public good—the object of their formation so frequently forgotten by the individual components.

Probably the most important question in the whole domain of social hygiene is that of sex education. The difficulties to surmount are in direct proportion to the seriousness of the subject. There is no other question relating to social matters so hedged about, so covered up, and so surrounded by prejudice and long established customs as that which relates to sex matters. Though reproduction is the most important function in life, and is essential to life, it is a subject that is rarely discussed outside of medical circles. The taboo is in direct proportion to its importance. No matter how firmly we believe that any subject that is fundamental and vital to health and morals is a proper one for serious thought, we are met with the stubborn fact that even educated people will not tolerate its discussion. This prejudice or fault in our education must give way to a nobler and purer sentiment. There are few now who deny the importance of educating the young in physiology and hygiene of sex; but there are very many who ask what constitutes sex-instruction, when should it be given, and by whom should it be taught? To be of any practical value to the young, it must be of such a nature and at such time as is necessary for the preservation of health, the development of right thinking, and to insure proper control. In ordinary education the subjects are so presented as to create interest by arousing the curiosity, but in sex matters a practically opposite course must be pursued, as the danger of arousing undue curiosity is great, for it is generally conceded that the less children and youth think of sex, the better. In the later years of adolescence, however, more direct instruction may be substituted. Whether the subject should be taught in the lower grades of our schools is debatable. In the higher schools and colleges it is necessary and proper, yet if we exclude the lower schools we are met with the proposition that the vast majority of children never pass to the high schools and colleges, and they must either receive instruction from some other source or not receive it at all. In the lower grades we are met with the second proposition that very few teachers are as yet properly trained to impart such instruction safely. We then, of course, fall back upon the parents. In many instances the parents are incompetent, and in others will not impart such instruction, and here again only the few receive instruction. We are, therefore, if we would make sex-instruction effective and reach those most in need of such instruction, compelled to fall back upon the public schools and train the teacher to take the place of the parents in many instances, and to supplement their work in others. The soil must be prepared carefully, the seed carefully selected and the young plants protected, or that fertile field, the mind of the young, will grow the rankest thistles in place of the purest and most beautiful flowers.

The third and final problem, the social hygienists must attack is that of legislation, for the ultimate object for which we have sought information and have sought to use it for the public enlightenment is to secure the enactment of such legislation as will assure higher standards of social conduct. Precipitate and irrational legislation is not desired, but it must ultimately apply to (a) the living conditions of the masses, (b) the question of prostitution, (c) the sterilization of the unfit, and (d) the further regulation of marriage.

These questions are all of the first importance, as they lay at the very foundation of society. It is the old question of whether the house will be builded on the solid rock of unimpaired physical, mental and moral manhood and womanhood, or on the shifting sands of disease, debauchery and crime.

It is probably true that we cannot legislate morals into the people, but we are continually trying to legislate crime and misconduct out of them. It is also probably true that one-half the world knows nothing of the other half, but when that other half is furnishing three-fourths of the future population, it should be somebody's concern how and under what conditions the future citizen is born and raised. The rich and well-to-do know little of the hardships of life, and their children feel none of the struggle for life among the poor. In one State there are over 300,000 men who draw a salary of less than $7.50 a week, and in another State there are many thousands who draw a weekly stipend of $4.50 or less. There is one large manufacturing plant that yearly declares enormous dividends that pays its many hundred unskilled laborers less than $8.00 per week. In these days of high cost of living, it is an open question how the poorer people live. The answer is that they do not live, they exist, without proper food, without proper shelter and without proper protection from disease. It is said that misery loves company, and it is in these circumstances we find the large families growing up under circumstances of ill nourishment and bad environment, that tend to make weaklings and criminals, not men and women. Many of our large establishments are turning out large profits for their stockholders, and at the same time, by squeezing the makers of their incomes—the men behind the wheels—to a bare existence wage, are encouraging degeneracy, pauperism and crime. If our legislative bodies would turn aside from their arduous labors of trying to make the rich man richer, and spend a portion of such time and labor in endeavoring to correct conditions among the poor that are correctable, they might be rendering a much more valuable service to their country. There is certainly a wide field for social betterment. It is said the greatest asset of a State is its normal children. How can children born and reared in the environment of poverty and crime be normal? Is this proposition of no concern to society?

A second subject for legislation is prostitution. The day is past for temporizing. That plan and every other plan short of prohibition and penalization have failed. It is said such laws cannot be enforced. This is probably true to some extent. But when prostitution is made a crime and the participants treated as the thief, the highwayman and the murderer, and both sexes are held equally guilty and made to pay the same penalty, it will have a deterrent effect.

A third proposition that is calling for legislation and in some States has already received legislative attention, is the sterilization of the unfit. The individuals of both sexes, the degenerates, the moral perverts and others who by successive crimes against society become dangerous to society, should forfeit their rights to be considered men and women, and should not be permitted to increase the national load of incompetence and crime. The conditions that exist in some countries are simply unbelievable. In one country alone tens upon tens of thousands of this class—known in the eyes of the law—are permitted to propagate their kind without let or hindrance. It is a well known fact that the unfit reproduce at a much more rapid rate than the fit.

The fourth but not least important subject for legislation is that of marriage. Anyone who has been up against this proposition in our State Legislatures knows how difficult it is to get the legislative ear on this subject. No one denies the evidence produced, but—and that word "but" weighs more than all the evidence—is the time opportune. One minister declared legislation to prevent the marriage of the unfit and the diseased was opposed to God's law. Another said that when he attempted to discuss the subject with his charge he was asked to desist. Political expediency, ignorance and prejudice thus thwart the best ideas of the intelligent. But the legal battle has only begun. It will be carried on until right and honor win, or some other and better way appears.

These are the problems of social hygiene. The first already has its answer, the second and the third are now working towards solution. God speed the day when intelligence shall safeguard the future.

